# Pulsatile low shear stress increases susceptibility to endothelial inflammation via upregulation of IFT and activation of YAP

**DOI:** 10.1063/5.0263936

**Published:** 2025-06-11

**Authors:** Yu Hou, Hazel R. C. Screen, Martin M. Knight

**Affiliations:** 1 Centre for Bioengineering, School of Engineering and Materials Science, Queen Mary University of London, London, United Kingdom; 2 Centre for Predictive in vitro Models, Queen Mary University of London, London, United Kingdom

## Abstract

This study describes the development of a microfluidic chip model of the coronary artery endothelium and its use to examine the mechanism through which pulsatile shear stress regulates inflammation. The chip successfully recapitulates increased susceptibility to cytokine mediated arterial inflammation as observed *in vivo* in areas of low shear stress (LSS). Previous *in vivo* data show that low shear stress in the porcine aorta modulates 36 cilia-associated genes of which five are also Yes‐associated protein (YAP) target genes. We demonstrate that pulsatile low shear stress (LSS) compared to high shear stress (HSS) preferentially drives YAP nuclear translocation and expression of the YAP target gene, Myosin Heavy Chain 10 (MYH10), which is also one of the cilia genes regulated by shear stress *in vivo*. LSS also increases expression of the cilia intraflagellar transport protein gene, IFT88, resulting in an increase in the primary cilia length and prevalence. Using a combination of siRNA and pharmaceutical regulators, we show that these changes in YAP, IFT88, and MYH10 drive the increased susceptibility to pro-inflammatory cytokines caused by LSS. Hence, we demonstrate that pulsatile LSS primes endothelial cells, increasing susceptibility to inflammation, and that this occurs through a novel pathway involving modulation of YAP and primary cilia/IFT. Such changes may also influence other cilia and YAP dependent responses. In conclusion, our microfabricated endothelial chip model reveals involvement of mechanosensitive IFT and YAP in arterial inflammation, which may provide novel therapeutic targets for the management of vascular disease such as atherosclerosis.

## I. INTRODUCTION

The etiology of arterial diseases, such as atherosclerosis, is associated with localized chronic inflammation. Endothelial cells (ECs) are exposed to a complex dynamic mechanical environment consisting of fluid shear stress caused by blood flow and associated pulsatile stretch of the vessel wall.^1^ Hemodynamic factors at the local level play a crucial role in influencing the development of atherosclerotic plaques.^2–4^ In particular, atherosclerosis is more prominent in human arterial regions where shear stress is below 10 dynes/cm^2^, typically associated with more turbulent flow around bifurcations. Conversely, straight arterial segments with laminar flow experience shear stress levels of 12–15 dynes/cm^2^ and appear to be less susceptible to inflammation.^5–8^ In areas of inflammation, pro-inflammatory cytokines, such as TNF-α, activate pro-inflammatory signaling pathways, leading to the activation of transcriptional factors such as NF-κB, which regulate EC phenotype.^9^ This causes up-regulation of endothelial cell adhesion molecules, such as VCAM-1 and ICAM-1,^10^ facilitating monocyte–endothelial cell interaction.^11^ Thus, chronic, localized activation of pro-inflammatory signaling pathways involves a fine interplay between biomechanical and inflammatory stimuli, which regulates vascular health and disease.

Primary cilia are slender hair-like organelles covered with a specialist membrane, and extending from the surface of almost all mammalian cell types with a single cilium on each ciliated cell.^12^ These cilia are composed of an array of microtubule doublets, along which specialized motor proteins known as intraflagellar transport (IFT) proteins, function to transport cargo on and off the axoneme.

Primary cilia have been shown to be involved in a wide range of cell signaling pathways important in development, health, and disease.^13,14^ Primary cilia are believed to play a role in mechanotransduction in a range of cell types including endothelial cells, although the underlying mechanisms are still unclear.^15^ In addition, the expression of cilia is regulated by the mechanical environment, with reduced ciliogenesis caused by tensile strain,^16^ compression, and fluid shear.^17^ Within the artery, elongated cilia are present on endothelial cells in regions exposed to low shear stress (LSS) or disturbed blood flow.^18,19^ In contrast, cilia are typically absent from endothelial cells in areas exposed to high laminar flow.^20^ One of the putative mechanisms for mechano-regulation of cilia expression identified in other cell types, involves the YAP pathway.^21,22^

Yes‐associated protein (YAP), a Hippo signaling pathway effector, is one of the most sensitive transcription factors activated in response to mechanical stress.^23,24^ Studies have shown that altered mechanical stimuli, including extracellular matrix stiffness, can regulate YAP nuclear localization by altering cell contractility.^25^ Within endothelial cells, YAP activity is regulated by flow and shear stress, both *in vitro* and *in vivo*.^26,27^ Myosin Heavy Chain 10 (MYH10), one of YAP target genes,^28^ is reported up-regulated by low shear stress *in vivo*.^29^ MYH10 is also a cilia-associated gene as defined in Cilia Carta.^30^ Figure S1 shows data from a previous *in vivo* study of flow sensitive genes in the porcine aorta,^29^ re-analyzed to highlight cilia-associated genes as well as YAP target genes.

Interestingly, understanding of cilia associated signaling continues to expand and now also includes a role in inflammatory signaling. Previous studies have shown that depletion of primary cilia associated intraflagellar transport IFT88 by hypomorphic mutation and siRNA, both disrupt pro-inflammatory signaling.^31,32^ Separate studies have also shown that this abolishes the anti-inflammatory effects of mechanical loading.^33^ Considering that IFT88 emerges as the top-ranked gene in the CiliaCarta database,^30^ which systematically catalogues ciliary genes, we therefore selected IFT88 as the primary focus of this study.

However, no studies have investigated whether the exacerbated arterial inflammation in areas of pulsatile LSS is mediated by alterations in YAP and IFT or primary cilia expression. Thus, we hypothesis that endothelial primary cilia, and/or IFT, are necessary for pro-inflammatory signaling in the artery. We hypothesis that pulsatile high shear stress (HSS) disrupts IFT and associated ciliogenesis, via a YAP dependent mechanism, thereby protecting most of the artery, while LSS triggers localized IFT/cilia expression, pre-disposing these areas to inflammation and initiation of atherosclerosis.

To evaluate this novel hypothesis, we developed a microfluidic arterial endothelial chip model of the human coronary artery and examined the role of YAP and primary cilia/IFT in the regulation of pro-inflammatory susceptibility to TNF-α by LSS. We demonstrated that arterial inflammation is exacerbated in areas of LSS and this via a YAP and IFT/primary cilia dependent mechanism. This suggests that inhibition of these mechanosensitive YAP or IFT responses may have therapeutic potential for vascular disease.

## II. RESULTS

### A. LSS increases nuclear YAP and IFT/primary cilia expression compared to HSS

**FIG. 1.**
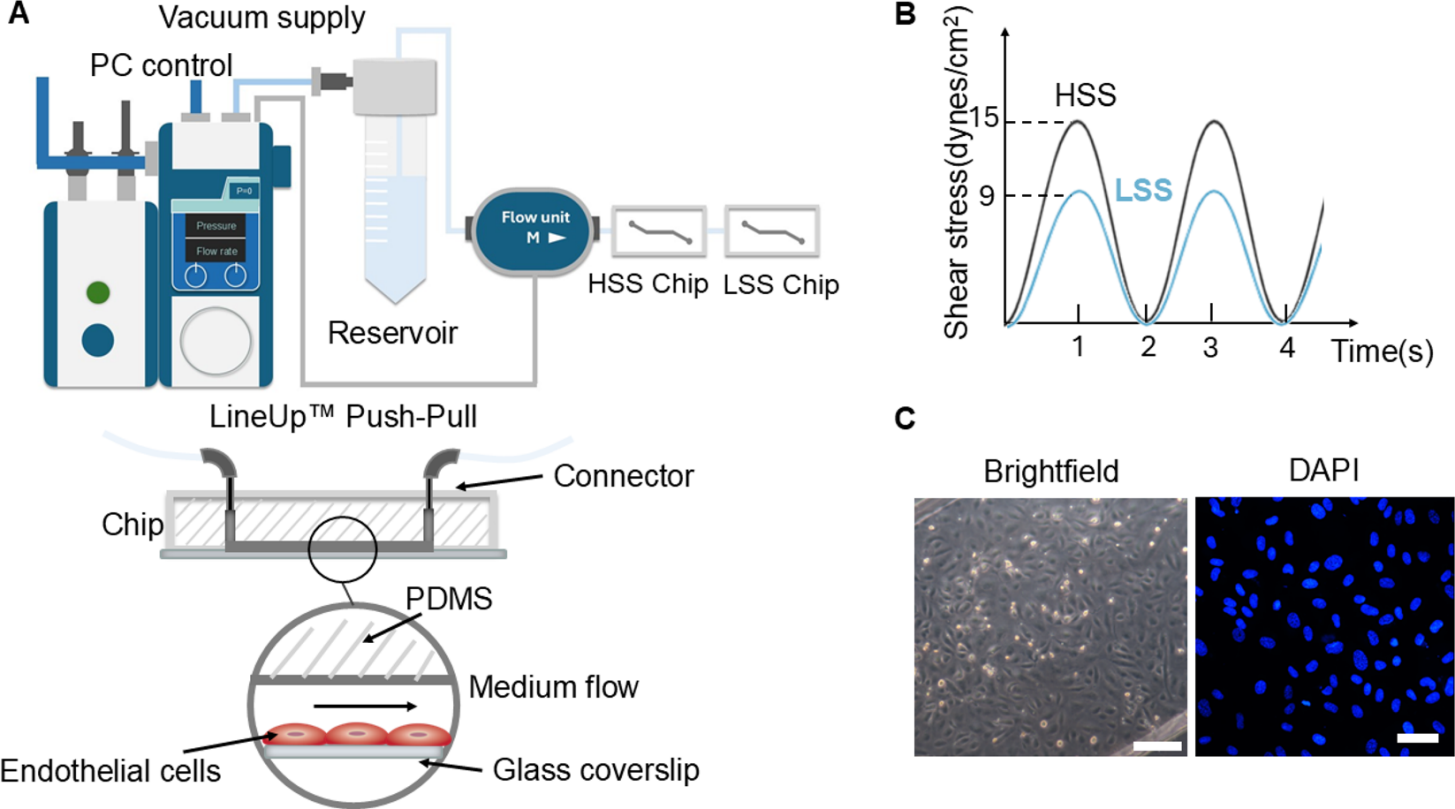
Arterial endothelial model with controlled pulsatile physiological flow. (a) Schematic showing the cell-seeded PDMS chips with glass coverslip bottom seeded with coronary artery endothelial cells and connected to a precisely controllable flow system from Fluigent. (b) A high shear stress (HSS) chip with a channel height of 100 *μ*m and a low shear stress (LSS) chip with a channel height of 125 *μ*m were connected in series. This enabled the application of two different pulsatile shear stress protocols at a frequency of 0.5 Hz and a peak shear stress of either 15 dynes/cm^2^ (HSS) or 9 dynes/cm^2^ (LSS). (c) Representative microscopy images of HCAECs in the chip obtained using brightfield (left, scale bar = 100 *μ*m) and fluorescence imaging of DAPI stained nuclei (right, scale bar = 50 *μ*m).

We successfully developed a simple microfluidic arterial endothelial model based on photolithography of a polydimethylsiloxane (PDMS) single channel chip (Fig. S2). The chips were seeded with human coronary artery endothelial cells (HCAECs) and subjected to pulsatile flow of either 0–15 dynes/cm^2^ (high shear stress, HSS) or 0–9 dynes/cm^2^ (low shear stress, LSS) as shown in Fig. 1. After 2 days culture on collagen and fibronectin, 4 h of pulsatile shear stress induced no detectable cell detachment. There was also no difference in cell density between LSS and HSS conditions with mean values (±SD) of 66.3 (±5.1) and 59.0 (±5.8) × 10^3^ cells/cm^2^, respectively (p > 0.05). There was also no difference in nuclear morphology with mean aspect ratios of approximately 1.5 for all groups (Fig. S3). Neither LSS nor HSS induced any nuclear reorganization relative to the axis of flow when compared to no shear stress control (Fig. S3).

**FIG. 2.**
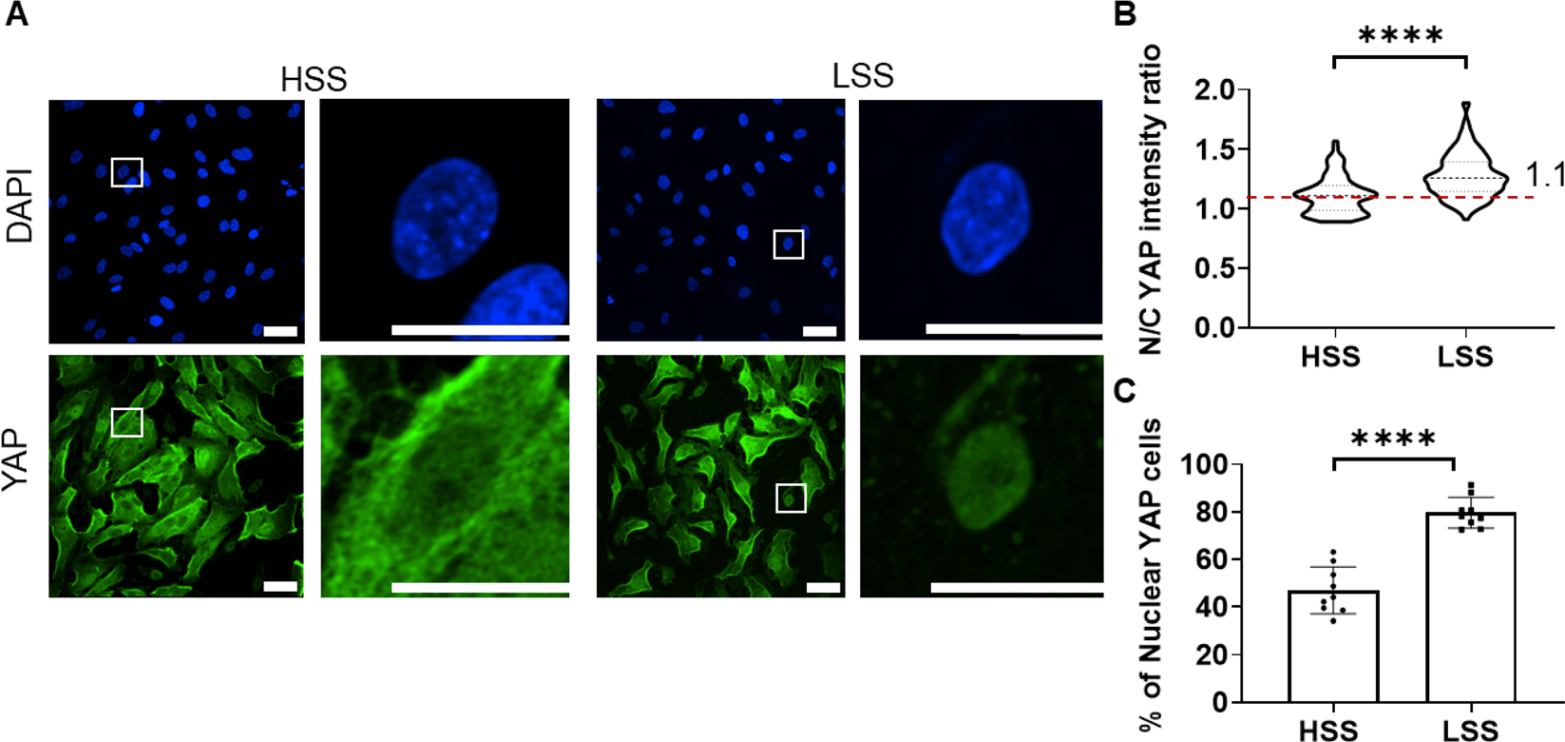
Endothelial cells exposed to LSS exhibit a greater YAP expression compared to cells exposed to HSS. Cells were subjected to HSS or LSS for 4 h prior to fixation. (a) Representative confocal images of YAP (green) and nucleus (blue). Scale bar = 40 *μ*m. (b) Corresponding nuclear/cytoplasmic (N/C) YAP staining intensity ratios based on CellProfiler software (n = 200 cells). The dashed line indicates the threshold of 1.1, used for defining the number of nuclear YAP positive cells (see also Fig. S10). (c) Corresponding percentage of cells in which the N/C YAP staining ratio was above the threshold of 1.1 (n = 9 fields). Bars represent mean ± SD. Data from three independent experiments. Statistical analysis was based on Mann–Whitney U test (b) and Student t test (c).

Cells exposed to LSS showed greater levels of nuclear YAP expression compared to those cells exposed to HSS, based on confocal immunofluorescence [Fig. 2(a)]. This was quantified by the ratio of nuclear to perinuclear cytoplasmic intensity of YAP staining with significantly higher ratios following LSS compared to HSS [Fig. 2(b)]. Similarly, LSS resulted in a significantly greater percentage of cells in which the ratio of nuclear to cytoplasmic YAP staining intensity was greater than the optimized threshold of 1.1 [Fig. 2(c)].

**FIG. 3.**
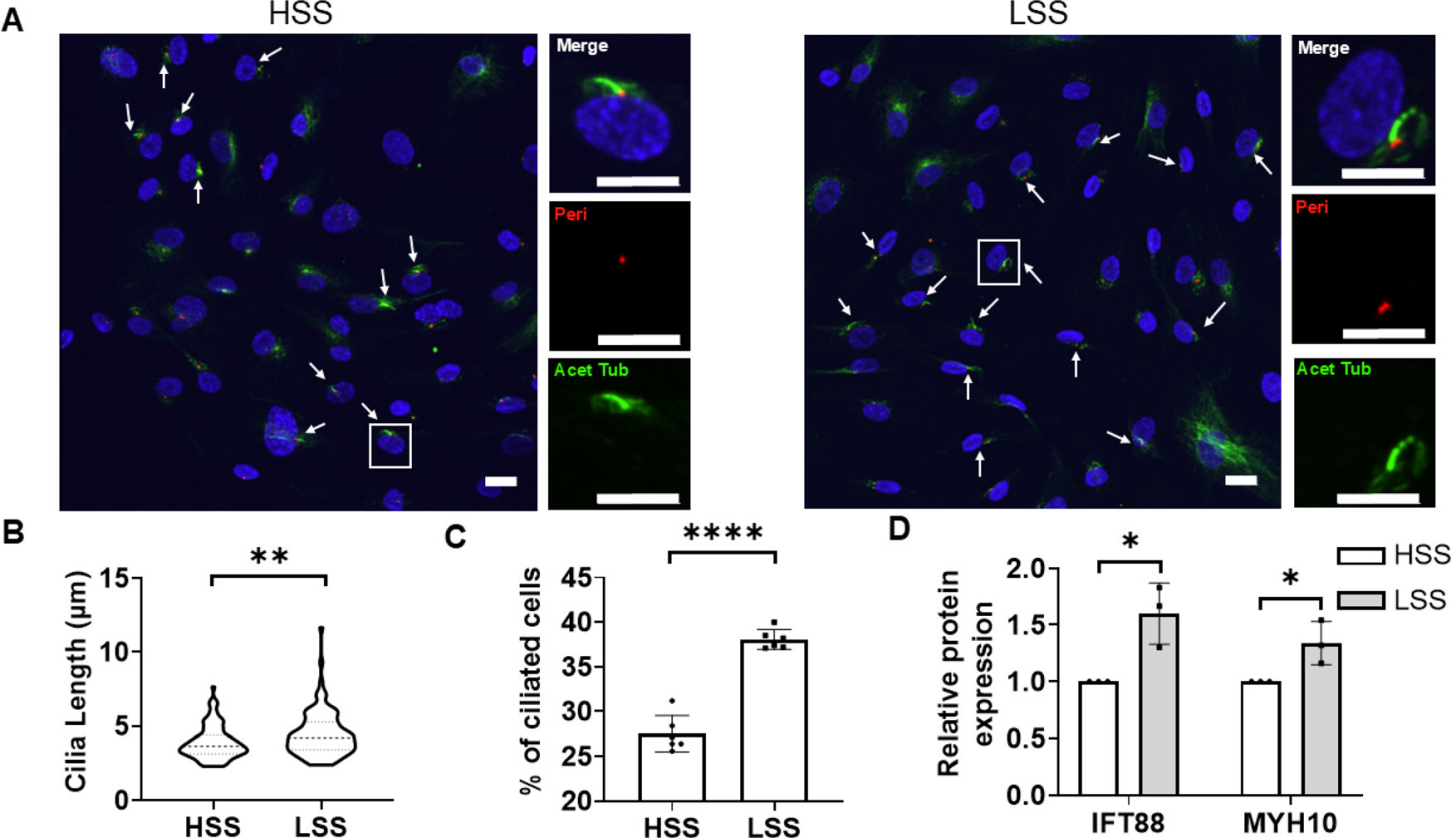
Endothelial cells exposed to LSS exhibit a greater IFT/primary cilia expression compared to cells exposed to HSS. Cells were subjected to HSS or LSS for 4 h prior to fixation or processing for Western blot. (a) Representative confocal images of primary cilia showing the axoneme and basal body labeled using anti-acetylated α-tubulin (green) and anti-pericentrin (red), nucleus (blue). Scale bar = 20 *μ*m. (b) Corresponding cilia length (n = 100 cilia) and (c) the percentages of ciliated cells (n > 600 cells). (d) Relative protein expression of MYH10 and IFT88 in LSS groups compared with HSS groups (n = 3). Data from three independent experiments. Bars represent mean ± SD. Statistical analysis was based on Mann–Whitney U test for (b) (p = 0.0026) and Student t test (c) and (d).

HCAECs expressed elongated primary cilia identified by confocal immunofluorescence labeling of the axoneme and basal body with antibodies to acetylated α-tubulin and pericentrin, respectively [Fig. 3(a)]. Cells exposed to 4 h HSS expressed cilia with a mean length of 3.9 *μ*m and prevalence of 27%. By contrast, cells exposed to LSS had a small, but statistically significant increase in cilia length [Fig. 3(b)] and a significant increase in prevalence to 38% [Fig. 3(c)]. This increase in cilia length and prevalence in LSS conditions was associated with an increase in the expression of intraflagellar transport protein, IFT88, which is necessary for cilia assembly [Figs. 3(d) and S4]. There was also an increase in the expression of the cilia-associated protein, MYH10 [Figs. 3(d) and S4]. MYH10 is also a YAP target gene, thereby confirming the activation of YAP in agreement with increased nuclear translocation (Fig. 2).

These results demonstrate that LSS activates YAP with associated increase in the expression of MYH10, and LSS increases the expression of IFT88 with associated increase in primary cilia length and prevalence.

### B. LSS increases pro-inflammatory sensitivity to TNF-α relative to HSS

**FIG. 4.**
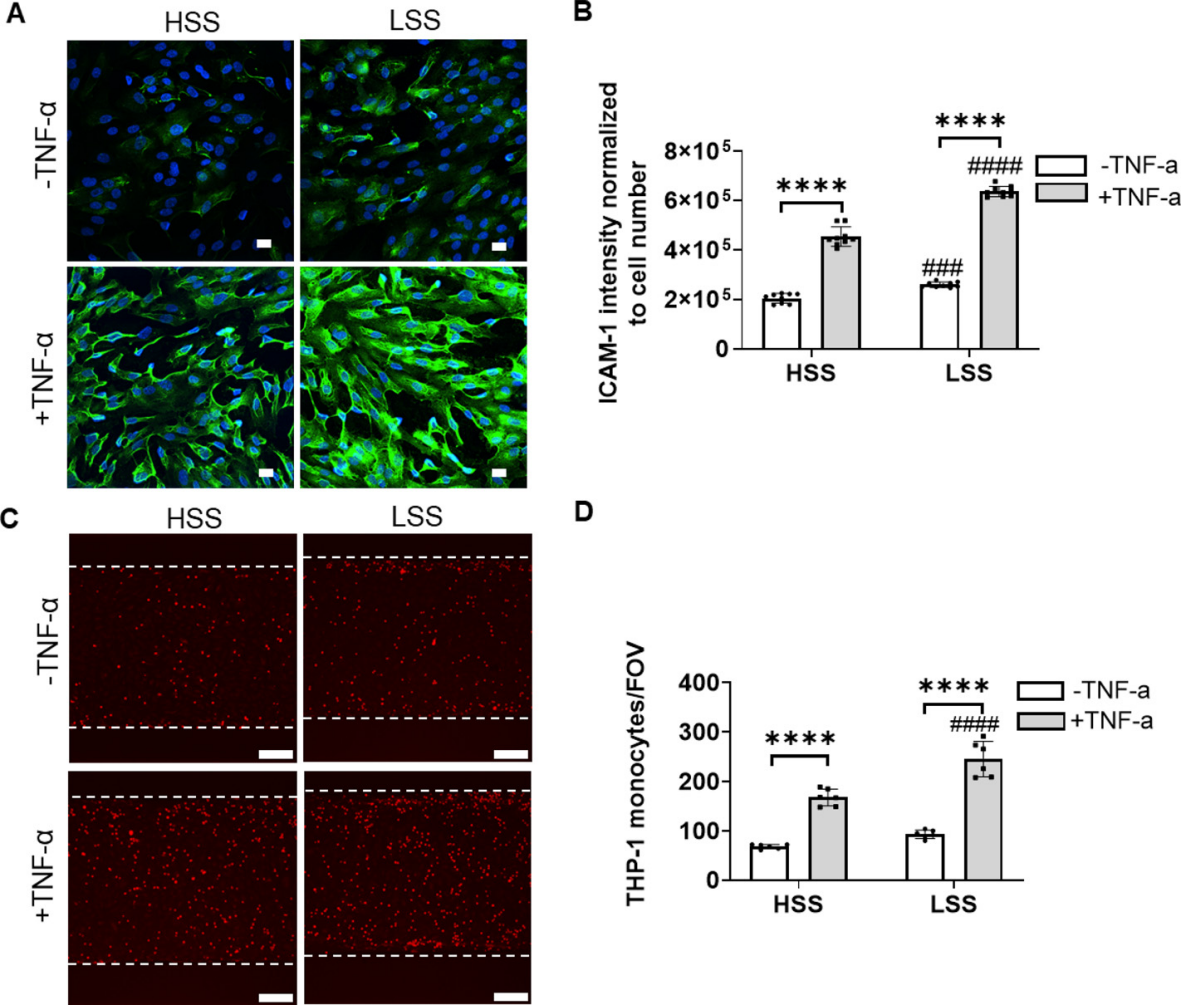
Endothelial cells exposed to LSS exhibit a greater ICAM-1 expression and THP-1 monocytes attachment with and without TNF-α compared to cells exposed to HSS. Cells were subjected to 4 h pulsatile flow at either HSS or LSS, followed by a further 4 h ± TNF-α (20 ng/ml) prior to fixation (a, b) or adding the THP-1 monocyte (c, d). (a) Representative confocal images of ICAM-1 (green) and nucleus (blue). Scale bar = 20 *μ*m. (b) ICAM-1 integrated intensity normalized to cell number (n = 9 fields). In separate experiments, fluorescently labeled THP-1 monocytes were added to the cells and maintained for 2 h under no flow conditions prior to washing. (c) Representative images of resulting THP-1 monocytes adhesion (red) to the HCAECs. Scale bar = 200 *μ*m. (d) Quantification of monocyte attachment (n = 6 fields). Data from three independent experiments. In all cases, bars represent mean ± SD. Statistical analysis was based on a two-way ANOVA with Bonferroni post hoc indicated ± TNF-α (*) and between HSS and LSS (^#^).

To investigate whether mechanical stimuli in the form of shear stress, regulates endothelial susceptibility to inflammatory cytokines, we tested the effects of LSS and HSS on ICAM-1 expression induced by TNF-α. In the absence of shear stress, stimulation of HCAECs with TNF-α (20 ng/ml) produced a small but statistically significant increase in ICAM-1 expression after just 2 h (Fig. S5). However, ICAM-1 staining intensity increased twofold after 4 h exposure, and threefold after 6 h, with sustained increases up to 10 h of exposure. Therefore, to measure the effect of shear stress, a protocol was adopted such that HCAECs were cultured for 2 days without flow and then exposed to 4 h pulsatile HSS or LSS, followed by a further 4 h with the same level of shear stress ± TNF-α (20 ng/ml). Without TNF-α, cells exposed to LSS showed a small increase in ICAM-1 expression compared to those exposed to HSS (Fig. 4). When shear stress and TNF-α were combined, a much larger increase in ICAM-1 expression was evident as expected. However, the increase was significantly greater under LSS than HSS conditions [Figs. 4(a) and 4(b)]. The fluorescently labeled THP-1 monocytes were subsequently added into the channel after the cells exposed to pulsatile HSS or LSS, with or without TNF-α (20 ng/ml). TNF-α increased subsequent adhesion of fluorescently labeled THP-1 monocytes onto the HCAECs; however, this adhesion was greater following LSS compared to HSS, as quantified by statistically significant differences in the number of adhered monocytes per field of view [Figs. 4(c) and 4(d)].

Together, the results demonstrate that LSS compared to HSS, primes or sensitizes arterial endothelial cells such that they show a greater inflammatory response, and associated monocyte adhesion, when exposed to TNF-α.

### C. Upregulation of inflammatory signaling by LSS in the presence of TNF-α, is dependent on YAP and IFT

Having confirmed the pro-inflammatory effect of pulsatile LSS relative to HSS, in priming HCAECs to be more susceptible to TNF-α, we next sought to understand the mechanisms involved.

To examine the involvement of YAP and IFT modulation in shear stress induced changes in pro-inflammatory susceptibility, we knocked down YAP and IFT using small interfering ribonucleic acid (siRNA). We then measured the inflammatory response to TNF-α in the absence of fluid shear stress. Cells were treated with siRNA for 5 hrs, followed by a further 4 h treatment with ± TNF-α (20 ng/ml). Analysis of pro-inflammatory response was based on both ICAM-1 expression, and nuclear expression of the NF-κB signaling protein, P65.

**FIG. 5.**
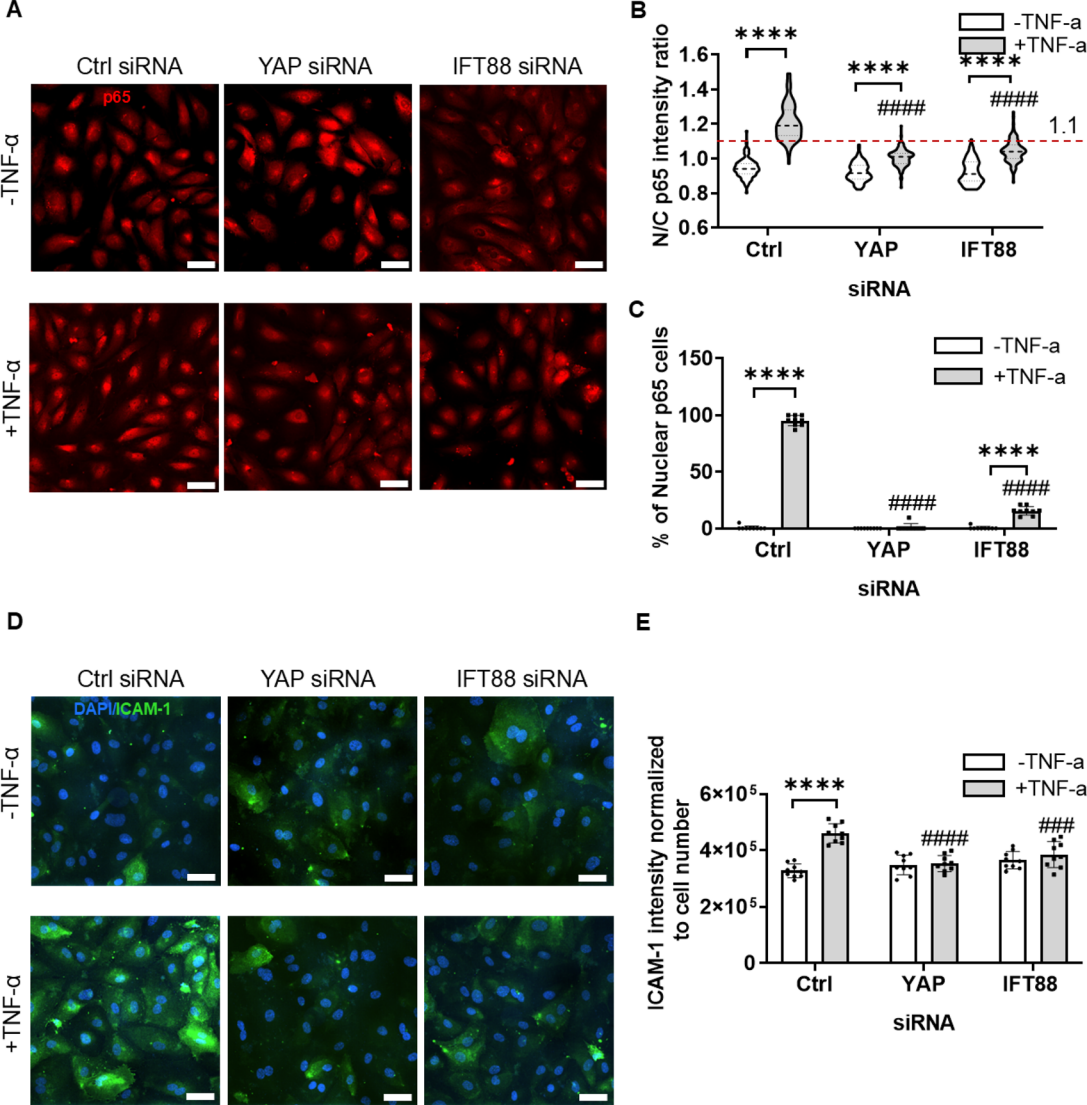
Endothelial inflammation caused by TNF-α is blocked by siRNAs to YAP and IFT88. Cells were treated with either YAP siRNA, IFT88 siRNA, or Ctrl siRNA for 5 h, followed by a further 4 h of treatment with ± TNF-α (20 ng/ml) prior to fixation and analysis. (a) Representative confocal images of P65 (red) and (b) corresponding nuclear to cytoplasmic (N/C) ratio of P65 staining intensity (n = 100 cells) and (c) percentage of nuclear positive P65 cells in which the N/C ratio was greater than 1.1 [dashed line in (b)] (n = 9 fields). (d) Confocal images of ICAM-1 (green) counterstained for nuclei (blue) and (e) corresponding ICAM-1 integrated intensity normalized to cell number (n = 9 fields). Bars represent mean ± SD with data from three independent experiments. Scale bars = 50 *μ*m. Statistical analysis was based on a two-way ANOVA with Bonferroni post hoc indicated ± TNF-α and between YAP siRNA, IFT88 siRNA relative to Ctrl siRNA. Statistical analysis was based on a two-way ANOVA with Bonferroni post hoc indicated ± TNF-α (*) and between YAP siRNA, IFT88 siRNA relative to Ctrl siRNA (^#^).

Knockdown was confirmed by Western blotting (Fig. S6). In control cells with scrambled siRNA, treatment with TNF-α significantly increased both nuclear translocation of P65 [Figs. 5(a)–5(c)] and ICAM-1 intensity [Figs. 5(d) and 5(e)]. By contrast, knockdown of YAP and IFT88 with siRNA reduced both these inflammatory responses to TNF-α (Fig. 5).

**FIG. 6.**
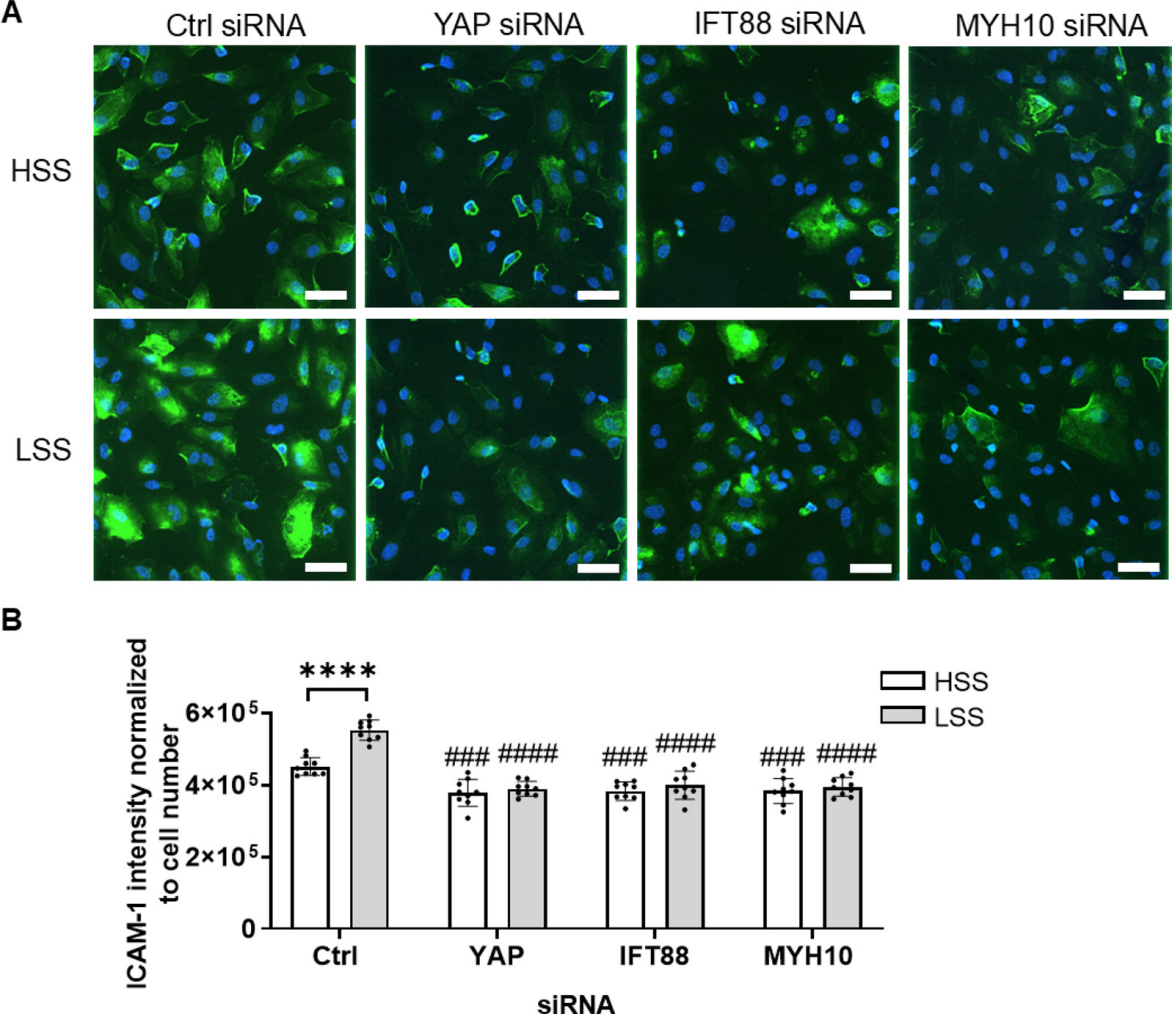
Endothelial inflammation caused by LSS is blocked by siRNAs to YAP, MYH10, and IFT88. Cells were treated with siRNAs to either YAP, IFT88, MYH10, or scrambled control for 5 h, followed by a further 4 h pulsatile flow at either HSS or LSS in the presence of TNF-α (20 ng/ml) prior to fixation and analysis. (a) Confocal images of ICAM-1 (green) counterstained for nuclei (blue) and (b) corresponding ICAM-1 integrated intensity normalized to cell number (n = 9 fields). Bars represent mean ± SD with data from three independent experiments. Scale bars = 50 *μ*m. Statistical analysis was based on a two-way ANOVA with Bonferroni post hoc indicated between LSS and HSS (^*^) and between YAP, IFT88, and MYH10 siRNAs relative to Ctrl siRNA (^#^).

Further siRNA studies were then conducted for cells subjected to LSS and HSS within the microfluidic chip. In addition to YAP and IFT88, we also knocked down the YAP reporter gene and cilia gene, MYH10, which has been shown to be regulated by shear stress *in vivo* (Fig. S1).^29^ In control cells transfected with scrambled siRNA, TNF-α treatment for 4 hrs increased ICAM-1 expression, with significantly greater response under LSS conditions compared to HSS conditions (Fig. 6). This matched the behavior seen in non-transfected cells (Fig. 4). Importantly, knockdown using siRNAs to YAP, IFT88 and MYH10 eliminated this differential effect, such that there were no significant differences in ICAM-1 expression between LSS and HSS conditions. As well as a major reduction in the ICAM-1 response to LSS, all three siRNAs also slightly reduced the inflammatory responses to HSS when compared with scrambled siRNA. These results demonstrate that YAP, IFT88, and MYH10 mediate the increased inflammatory response to LSS.

In various other cell types, lysophosphatidic acid (LPA) is known to activate YAP leading to its increased nuclear translocation.^34^ Thus, we examined whether LPA could activate YAP in HCAECs and whether this was associated with increased inflammatory susceptibility to TNF-α. LPA (100 *μ*M) activated YAP signaling after 1 h of treatment with a sustained response at 4 and 6 h (Fig. S7). Additional studies found that treatment with LPA for 4 h produced a small but significant increase in the expression of the pro-inflammatory marker, ICAM-1 relative to no LPA controls, with no effect on cell viability or proliferation (Fig. S8). However, interestingly in the presence of TNF-α, LPA had minimal effect on YAP activation (Fig. S8) and actually reduced the expression of the pro-inflammatory marker, ICAM-1. This suggested that this anti-inflammatory effects of LPA on HCAECs may occur through a separate, YAP-independent pathway.

### D. Inter-dependency between YAP and IFT/primary cilia expression in HCAECs

We have shown that LSS increases inflammatory susceptibility relative to HSS, and that this is dependent on the expression of YAP and IFT88 and associated with increased cilia length and prevalence. We next set out to untangle the relationship between YAP and IFT88/cilia to identify whether one is upstream of the other in the mechanosensitive cascade that modulates sensitivity to TNF-α. To do this, we again treated with siRNAs to either YAP or IFT88 and in both cases measured the effects on YAP and cilia expression using confocal immunofluorescence.

**FIG. 7.**
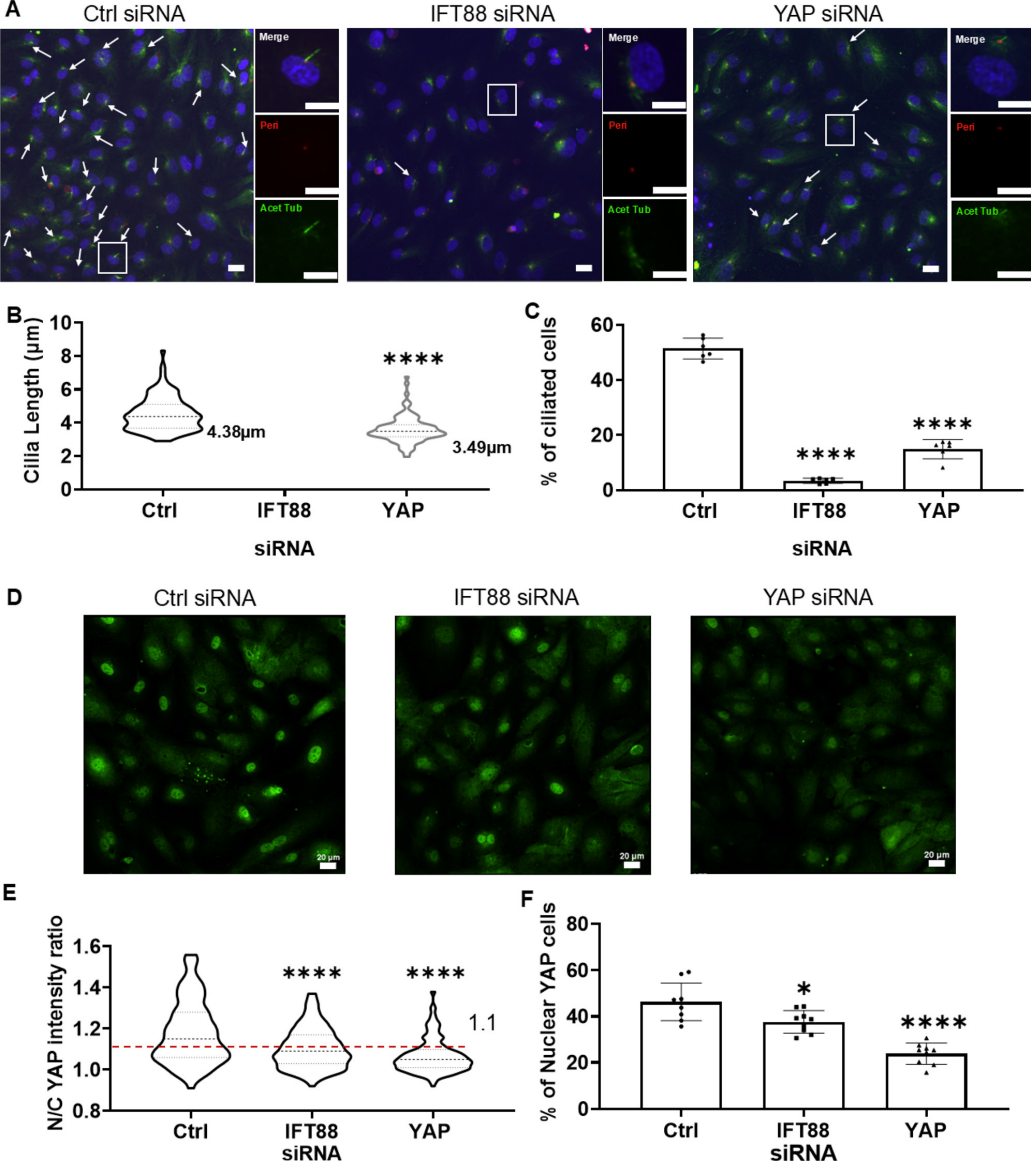
Inter-dependency between YAP and IFT88/primary cilia expression. Cells were treated with either YAP siRNA, IFT88 siRNA, or Ctrl siRNA for 5 hrs prior to fixation. (a) Representative confocal images of primary cilia (arrows) showing axoneme and basal body labeled using anti-acetylated α-tubulin (green) and anti-pericentrin (red), nucleus (blue) and (b) corresponding cilia length (n = 100 cilia) and (c) the percentage of ciliated cells (n > 600 cells). (d) Representative confocal images of YAP (green) and (e) corresponding nuclear/cytoplasmic (N/C) YAP intensity ratios (n = 200 cells) and (f) the percentage of nuclear YAP positive cells in which the N/C ratio was greater than 1.1 (dashed line in E). Scale bar = 20 *μ*m. Data from three independent experiments. Bars represent mean ± SD. Statistical analysis was based on one-way ANOVA test with differences shown relative to control group.

As expected, treatment with siRNA to IFT88 abolished ciliogenesis such that only 4% of HCAECs exhibited a primary cilium and these were so short and stunted that length measurements were not possible [Figs. 7(a)–7(c)]. Disruption of IFT88 also reduced YAP protein expression (Fig. S6) and nuclear localization of YAP [Figs. 7(d)–7(f)]. Conversely, YAP siRNA reduced YAP N/C ratio as expected (Figs. 7(d)–7(f)], but also reduced cilia length and prevalence [Figs. 7(a)–7(c)] and partially reduced IFT88 protein expression (Fig. S6).

Together, these data indicate significant bidirectional interaction between YAP and IFT which both drive the increased inflammatory susceptibility in HCAECs exposed to LSS relative to HSS.

## III. DISCUSSION

In the present study, we developed a simple human coronary arterial endothelial chip model and showed that this could replicate the enhanced susceptibility to inflammation in areas of pulsatile low shear stress (LSS) compared to high shear stress (HSS). We then used this model to examine the underlying mechanism and reveal the complex involvement of YAP, IFT signaling, and associated primary cilia assembly.

The simple arterial endothelial chip model was successfully fabricated from PDMS using photolithography and connected to a Fluigent pump system providing precisely controlled pulsatile fluid flow. A glass coverslip was bonded to the base successfully enabling high resolution confocal microscopy of the endothelial cells.

The alignment of endothelial cells in response to fluid shear stress is somewhat cell type dependent with aortic valve endothelial cells aligning perpendicular to flow,^35^ while the majority of studies with vascular endothelial cells show alignment parallel to steady flow.^36^ This cellular alignment parallel to flow occurs through a mechanism involving VEGF signaling and Src family pathway activation.^37^ By contrast, in the present study, coronary artery endothelial cells exposed to 8 h of pulsatile flow at either high or low shear stress, showed minimal reorientation response to flow in any direction, retaining the even distribution of directionality seen without flow (Fig. S3). One explanation for the difference is that the dynamic shear stress environment generated using physiological pulsatile flow, as adopted in the present study, may lead to less consistent cell alignment compared to steady flow. Indeed, using the same system, we demonstrate cell alignment parallel to flow in response to non-pulsatile steady shear stress (Fig. S9). Endothelial cells *in vivo* are aligned parallel to the long axis of the artery and hence the direction of pulsatile flow.^37^ However, this *in vivo* organization may be due, at least in part, to cells aligning perpendicular to the direction of the circumferential tensile strain as reported in numerous *in vitro* studies.^38,39^ Therefore, to more accurately mimic the *in vivo* biomechanical environment and associated cell morphology, future arterial endothelial chip models should ideally incorporate both pulsatile fluid shear stress and perpendicular cyclic tensile strain.^39–41^

Interestingly, in the presence of TNF-α and high shear stress (HSS), cells preferentially realigned perpendicular to the axis of pulsatile flow (Fig. S3). It is not clear why the pro-inflammatory cytokine should induce this cellular reorientation to HSS. However, TNF-α is well known to induce a more stressed cell state with increased actin tension, expression of adhesion complexes, and migration, all of which are likely to play a role in cellular realignment to shear stress.^42^

In the present study, HSS was associated with a lower expression of elongated primary cilia compared to LSS, as characterized by reduced cilia length and prevalence (Fig. 3). This is consistent with *in vivo* findings where regions of the artery exposed to high shear stress and laminar flow show reduced expression of primary cilia compared to areas with lower shear stress^14^ and more complex flow.^20^ For example, Dinsmore and Reiter showed that cilia are more common on the inner curvature of the aortic arch, compared to the outer curvature where shear stress is greater.^19^

The mechanism for shear stress-induced loss of cilia in endothelial cells remains unclear. In other cell types, loss of cilia has been mediated by HDAC6 and TGFβ in response to various mechanical stimuli including cyclic tensile stretch and compression.^33,43^ Further studies have shown that YAP activation, which occurs in response to mechanical stimuli,^26,27^ may trigger a resorption of primary cilia through increased actin tension.^44^ However, our data showed the opposite, first that YAP is preferentially activated by LSS compared to HSS (Fig. 2) and second, that this is associated increased cilia expression rather than resorption (Fig. 3). Our data are nevertheless consistent with findings that endothelial cells exposed to low shear, disturbed flow, show a greater YAP activation and nuclear translocation compared to high shear stress, laminar flow.^26^ Interestingly the YAP activation with LSS observed in our study is not only greater than with HSS, but it is also greater than in static no flow conditions (Fig. S7). This further highlights the need for arterial endothelial chip models to recreate the true physiological biomechanical environment in order to replicate the *in vivo* biological responses.

Ciliary assembly and disassembly is dependent on microtubule-based motor transport of tubulin on and off the axoneme using a bidirectional process termed intraflagellar transport (IFT). Previous studies have demonstrated that the knockdown of IFT88 through either siRNA or hypermorphic mutation, reduces cilia expression in various cell types.^45–47^ Our data demonstrate that siRNA to IFT88 also reduced endothelial cilia length and prevalence (Fig. 7). Furthermore, reduced cilia expression in response to HSS was associated with reduced expression of IFT88 at the protein level [Fig. 3(d)].

Previous *in vitro* studies of other types of endothelial cell also show that high shear stress reduces expression of cilia genes including the putative IFT component, CMG-1,^48^ and cilia related genes KLF4 and KLF2.^49^ To determine whether similar mechanoregulation of ciliary genes and YAP target genes occurs with altered fluid shear stress *in vivo*, we data-mined a study by Evans *et al.*^29^ In this previous study, RNA samples of endothelial cells isolated from high and low time-averaged wall shear stress (WSS) regions of the porcine aorta revealed 867 shear-responsive genes, with 479 upregulated and 388 downregulated in low vs high WSS conditions.^29^ These shear sensitive genes were compared using MATLAB (R2022b, Natick) with a comprehensive compendium covering 956 human cilia genes published by Teunis *et al.* as “CiliaCarta”^30^ and a genomics gene set covering 2212 target genes of the YAP transcription factor.^28^ We identified 22 cilia genes that were upregulated by low shear stress and 14 genes that were downregulated (Fig. S1). Of the 22 cilia-associated genes, three were identified as YAP target genes (MYH10, MYO5B, and HYDIN), and among a separate set of 14 cilia-related genes, two YAP targets (GPR157 and GSK4B) were detected. MYH10 was the most strongly regulated by flow and hence was investigated in the present *in vitro* study. Here, we show that MYH10 was also upregulated under low shear stress compared to high shear stress [Fig. 3(d)] mimicking that seen *in vivo*. However, the observed upregulation of IFT88 under low shear stress using HCAECs *in vitro* was not replicated within the previous porcine *in vivo* study, possibly reflecting species differences between the two studies. However, other genes closely associated with cilia assembly were differentially upregulated in low shear stress conditions *in vivo*. For example, CCDC146, the third most upregulated gene in response to low shear stress *in vivo*, is known to interact with IFT88 such that knockout of CCDC146 decreases IFT88 protein levels.^50^

Not only does IFT regulate ciliogenesis, but it is also a key regulator of cilia signaling pathways including hedgehog, Wnt, and mechanosignalling.^15^ It is unclear whether the alterations in cilia formation and cilia gene expression caused by HSS impact on these cilia signaling pathways. Primary cilia and/or IFT have also been linked to pro-inflammatory NF-κB signaling induced by cytokines such as IL-1β and TNF-α.^32,51–53^ In the present study, the upregulation of cilia/IFT expression under LSS conditions relative to HSS was associated with increased pro-inflammatory susceptibility to TNF-α, based on ICAM-1 expression and monocytes attachment (Fig. 4).

In control cells, TNF-α treatment increased ICAM-1 expression, with significantly greater response under LSS conditions compared to HSS conditions (Fig. 4). The data also agree with previous *in vitro* studies, which show that low oscillating shear stress up-regulates both VCAM-1 and ICAM-1 compared to high static shear stress.^26^ These findings reflects the differences in endothelial inflammation seen *in vivo* between areas exposed to laminar high shear stress and lower oscillatory flow.^5,54^

In our study, the association between inflammatory signaling and expression of cilia/IFT and YAP, was confirmed using siRNAs to YAP, IFT88, and MYH10. In all three cases, knockdown eliminated the differential inflammatory response between LSS and HSS (Fig. 6). We therefore conclude that LSS preferentially pre-disposes coronary artery endothelial cells to the pro-inflammatory effects of TNF-α and that this occurs via a YAP and IFT88-dependent pathway. Interestingly, recent studies have also shown that modulation of pro-inflammatory macrophages also occurs via a YAP-dependent mechanism involving regulation of Spp1, IL6, and ARG1.^55^

The present study also examined the interplay between YAP and IFT/primary cilia in regulation of inflammation. Knockdown of YAP with siRNA reduced inflammatory susceptibility to TNF-α, and reduced ciliogenesis (Fig. 7) and partially reduced IFT88 expression (Fig. S6). Conversely, siRNA disruption of IFT88 and ciliogenesis reduced nuclear translocation of YAP (Fig. 7). Together this suggests that the increased inflammatory sensitivity caused by LSS involves bi-directional interaction between YAP activation and alterations in IFT88. However, whether IFT88 is a transcriptional target of YAP or whether their relationship is mediated through other signaling pathways is not yet confirmed. TAZ has been reported as important mediators of mechanical cues instructed by the cellular microenvironment.^56^ YAP/TAZ inhibition also suppresses JNK signaling and downregulates pro-inflammatory genes expression, thereby reducing monocyte attachment.^27^ However, the crosstalk between TAZ activation dynamics and pulsatile shear stress remains unclear.

Interestingly, both YAP inhibition with siRNA, and the YAP agonist LPA reduced the inflammatory response to TNF-α shown by reduced ICAM-1 expression (Fig. S8). However, in the presence of TNF-α, LPA failed to upregulate YAP. While many previous studies have reported that LPA upregulates NF-κB signaling (for review see Ref. 57) including increased ICAM-1 mRNA,^58^ other studies have shown anti-inflammatory effects in a variety of cell types.^22,59^ The present study suggests that the anti-inflammatory effects of LPA in arterial endothelial cells may be highly context dependent and occur through a YAP-independent pathway.

## IV. CONCLUSION

In conclusion, we have used microfabrication techniques to develop an arterial endothelial chip model of the human coronary artery with physiological pulsatile flow. This model effectively recapitulates the enhanced susceptibility to cytokine induced inflammation in areas exposed to low shear stress (LSS) compared to areas exposed to high shear stress (HSS). We use this model to examine novel underlying mechanisms involving IFT and YAP. We demonstrate that pulsatile low shear stress increases IFT88 and MYH10 expression, with associated upregulation of ciliogenesis and YAP activation. Furthermore, we show that these changes in YAP and IFT/cilia are responsible for mechanically priming endothelial cells, causing increased sensitivity to the inflammatory cytokine, TNF-α. These findings suggest that IFT and YAP may provide novel therapeutic targets for management of vascular inflammation and associated atherosclerosis.

## V. METHODS

### A. Method details

#### 1. Manufacture of chips

Microfluidic chips were fabricated from polydimethylsiloxane (PDMS) with a single, rectangular cross section channel with width of 1000 *μ*m. To enable different shear stress with identical flow rates, two different chip designs were made with a channel height of either 100 or 125 *μ*m. The flow channel area is 0.25 cm^2^. The manufacture of these custom designed chips is shown schematically in Fig. S2. The chip mold template was designed using Solidworks software (Concord, MA, US) and fabricated using standard photolithography. The channel design was transferred to silicon wafers using SU-8 Photoresist (MicroChem Inc.) and exposure to UV light (UVK3, 2s, 90 Mw/cm^2^). PDMS was mixed at a 9:1 ratio (w/w) of polymer to the curing agent (Sylgard 184, Dow Corning, CA, US), poured onto the wafers and cured at 65 °C. The cured PDMS was subsequently separated from the wafer, and holes (1 mm diameter) were cored for the inlet and outlet using biopsy punches. Each PDMS chip was bonded to a rectangular coverslip glass coverslip base to enable high resolution imaging of the cells subjected to fluid shear stress. Bonding of the coverslip was achieved using a plasma cleaner, after which the sealed chip was thoroughly rinsed with de-ionized water.

#### 2. Cell culture

Human Coronary Artery Endothelial Cells (HCAECs) were purchased from Promocell (C-12221, derived from a single donor, N = 1) and maintained in complete Endothelial Cell Growth Medium MV with 5% serum (v/v) (Promocell). This medium was supplied as a kit and consisted of Basal Medium (C-22220) and Supplement Mix (C-22120) with Endothelial Cell Growth Supplement (0.004 ml/ml), Epidermal Growth Factor (recombinant human, 10 ng/ml), and Heparin (90 *μ*g/ml). For stimulation of an inflammatory response, TNF-α (PeproTech) was added to the medium at a final concentration of 20 ng/ml. Hydrocortisone (Promocell, 1 *μ*g/ml) was added only to the medium without TNF-α. Penicillin-streptomycin (P4333, Sigma, 1% v/v) was added to all medium. In preparation for cell seeding, the channels were rinsed with PBS and coated with the extracellular matrix (ECM) proteins, Fibronectin (33 *μ*g/ml, C-43060, PromoCell) and calf skin Collagen (33 *μ*g/ml, C8919, Sigma-Aldrich), for 2 hrs at 37 °C, 5% CO_2_. Cells were seeded to the flow channel of the chip or cell plate using a pipette. THP-1 monocytes (Invivogen, Toulouse, France) were cultured in Roswell Park Memorial Institute (RPMI) medium (Gibco) supplemented with 10% (v/v) FBS.

#### 3. Application of fluid shear stress

The cell-seeded chips were connected individually via fluorinated ethylene-propylene (FEP) tubing to a commercial programmable pressure-driven flow system (Fluigent, France) enabling the application of pulsatile shear stress (Fig. 1). The magnitude of shear stress was set by controlling the flow rate using OxyGEN software (Fluigent, France) and based on the equation describing shear stress in a rectangular channel.^60^ In this study, we simulated the shear stress present in different regions of the coronary artery induced by blood flow.^40,61^ Pulsatile flow was applied at 0.5 Hz to the cells within the two different chip designs thereby generating low shear stress (LSS) and high shear stress (HSS) environments with values of 0–9 dynes/cm^2^ and 0–15 dynes/cm^2^, in chips with channel height of 125 and 100 *μ*m respectively (Fig. 1).

#### 4. Immunofluorescence staining and confocal microscopy

Cells were fixed with freshly defrosted 4% paraformaldehyde (PFA, 10 min, room temperature) and permeabilized with 0.5% Triton-X/PBS (5 min, room temperature). Subsequently, the samples were blocked for 1 h at room temperature with 20% Fetal Bovine Serum (FBS, F7524, Sigma), incubated overnight at 4 °C with primary antibodies, washed in 0.1%BSA/PBS (3 × 10 min), and incubated with secondary fluorescent-conjugated antibodies (Table I). Cell nuclei were counterstained with DAPI (D9542, Sigma). Confocal z-stack imaging was performed using a Nikon CSU-W1 SoRa Spinning Disk Confocal laser scanning microscope with a 40×/0.9/NA objective. Confocal z-series were obtained consisting of 10 sections with a nominal z-spacing of 0.6 *μ*m. Maximum intensity projections were created, and the images analyzed using ImageJ (NIH) and CellProfiler software.

**TABLE I.** Antibodies used for immunofluorescence staining (IF) and Western blotting (WB).

Target	Feature	Cat no./species	Company	Dilution
Acetylated α-tubulin	Cilia axoneme	T7451/mouse	Sigma Aldrich	1:1000 (IF)
IFT88	IFT protein	13967-1-AP/rabbit	Protein tech	1:1000 (WB)
ICAM-1 (CD54)	Inflammatory marker	ab2213/mouse	Abcam	1:1000 (IF)
YAP	YAP protein	sc-101199/mouse	Santa Cruz	1:500 (IF)
YAP	YAP protein	CS4912/rabbit	Cell signaling	1:1000 (WB)
Pericentrin	Basal body	ab4448/rabbit	Abcam	1:1000 (IF)
NF-kB (P65)	Inflammatory marker	ab32536/rabbit	Abcam	1:1000 (IF)
β-actin	WB loading control	ab8226/mouse	Abcam	1:2000 (WB)
MYH10	YAP target gene	sc-376942/mouse	Santa Cruz	1:100 (WB)
β-actin	WB loading control	PA1-46296/rabbit	Invitrogen	1:2000 (WB)
Alexa Fluor 488	Secondary antibody	A21202/mouse	Invitrogen	1:1000 (IF)
Alexa Fluor 555	Secondary antibody	A32794/rabbit	Invitrogen	1:1000 (IF)
IRDye 680RD	Secondary antibody	C71204-03/mouse	LI-COR	1:2000 (WB)
IRDye 800CW	Secondary antibody	C90129-05/rabbit	LI-COR	1:2000 (WB)
Alexa Fluor 488	Secondary antibody	A32731/rabbit	Invitrogen	1:2000 (WB)
Alexa Fluor 546	Secondary antibody	A-11018/mouse	Invitrogen	1:2000 (WB)

The quantification of cellular localization of YAP and P65 based on confocal immunofluorescence was performed using CellProfiler software (Broad Institute), based on methods previously described (Fig. S10).^21^ The ratio of nuclear to perinuclear cytoplasmic intensity was calculated for each cell. The quantification of nucleus orientation and aspect ratio was performed using ImageJ (Fig. S11).

Primary cilia length was measured using NIS-Elements based on methods previously described.^62^ Vertical cilia, as identified by the confocal z-series, were excluded manually due to reduced axial resolution. The percentage of ciliated cell was calculated from the ratio of ciliated cells over total cells. The quantification of ICAM-1 integrated intensity and cell count was performed using ImageJ. The raw data were analyzed using GraphPad Prism 8 (GraphPad Software Inc, CA).

#### 5. Western blotting

Cells were lysed on ice in RIPA lysis buffer (R0278, Sigma Aldrich), and the protein concentrations of supernatants were quantified using a bicinchoninic acid assay (BCA) kit (Thermo Scientific). Aliquots of each protein lysate (10–20 *μ*g) were loaded and subjected by SDS/PAGE. After electrophoresis, proteins were transferred to polyvinylidene difluoride (PVDF) membranes, followed by blocking and incubation with primary and secondary antibodies (Table I). The blots were then imaged using iBright imaging system (FL1500, ThermoFisher).

#### 6. Transfection

Gene knockdown was performed using siRNA and compared to scramble control siRNA (sc-37007, Santa Cruz Biotechnology) according to the manufacturer's protocols. For YAP disruption, cells were transfected with 10 *μ*M YAP siRNA (sc-38637, Santa Cruz Biotechnology). For IFT/primary cilia disruption, cells were transfected with 10 *μ*M IFT88 siRNA (sc-75329, Santa Cruz Biotechnology). For MYH10 disruption, cells were transfected with 10 *μ*M MYH10 siRNA (sc-61122, Santa Cruz Biotechnology). The efficiencies of YAP, MYH10, and IFT88 knockdown were determined by Western blotting analysis after 5 h.

#### 7. THP-1 monocyte recruitment assay

On day 7, THP-1 monocytes were labeled with 2 *μ*M cell tracker dye (CMRA orange, Thermo Fisher Scientific) for 15 min in serum-free medium at 37 °C. Cells were then centrifuged at 300 ×g for 5 min to form a pellet, washed with serum-free medium and then resuspended to a concentration of 2 × 10^6^ cells/ml. Fluorescently labeled monocytes were added into the channel and then incubated under static conditions for 2 h. The channel was then flushed with Endothelial Cell Growth Medium to remove unattached cells, and chips were imaged to assess monocyte recruitment using confocal microscopy. Labeled monocytes were counted using ImageJ.

### B. Experimental design and statistical analysis

HCAECs were seeded into the chips and cultured for 48 h in the absence of flow to allow the cells to settle and a confluent monolayer to develop. Individual chips were then exposed to flow at either HSS or LSS for a period of 4 h prior to either fixation for microscopy analysis, or cell lyses for subsequent Western blotting. For each experimental condition, a HSS and a LSS chip were connected in series as shown in Fig. 1(a). Statistical analysis and n values for each experiment are outlined in the figure legends with significant differences indicated at p < 0.05 (* or ^#^), p < 0.01 (** or ^##^), p < 0.001 (*** or ^###^), and p < 0.0001 (**** or ^####^).

Initial experiments were conducted to determine the relative effect of LSS and HSS on cell morphology, YAP, IFT, MYH10, and associated primary cilia expression, followed by the effect on inflammation induced by TNF-α. We then determined whether there was a link between shear stress modulation of YAP, IFT, and shear stress modulation of inflammation, using siRNAs to YAP, MYH10, and IFT88. This enabled us to also examine the crosstalk by quantifying the effect of YAP knockdown on IFT88 and cilia expression, and vice versa, the effect of IFT88 knockdown on YAP.

## SUPPLEMENTARY MATERIAL

See the supplementary material for additional data.

## Data Availability

The data that support the findings of this study are available from the corresponding authors upon reasonable request.
